# Regulation of Expression and Evolution of Genes in Plastids of Rhodophytic Branch

**DOI:** 10.3390/life6010007

**Published:** 2016-01-29

**Authors:** Oleg Anatolyevich Zverkov, Alexandr Vladislavovich Seliverstov, Vassily Alexandrovich Lyubetsky

**Affiliations:** Institute for Information Transmission Problems of the Russian Academy of Sciences (Kharkevich Institute), Bolshoy Karetny per. 19, Build. 1, Moscow 127051, Russia; slvstv@iitp.ru (A.V.S.); lyubetsk@iitp.ru (V.A.L.)

**Keywords:** plastid, protein, transcription factor, translation initiation, clustering

## Abstract

A novel algorithm and original software were used to cluster all proteins encoded in plastids of 72 species of the rhodophytic branch. The results are publicly available at http://lab6.iitp.ru/ppc/redline72/ in a database that allows fast identification of clusters (protein families) both by a fragment of an amino acid sequence and by a phylogenetic profile of a protein. No such integral clustering with the corresponding functions can be found in the public domain. The putative regulons of the transcription factors Ycf28 and Ycf29 encoded in the plastids were identified using the clustering and the database. A regulation of translation initiation was proposed for the *ycf24* gene in plastids of certain red algae and apicomplexans as well as a regulation of a putative gene in apicoplasts of *Babesia* spp. and *Theileria* *parva*. The conserved regulation of the *ycf24* gene expression and specificity alternation of the transcription factor Ycf28 were shown in the plastids. A phylogenetic tree of plastids was generated for the rhodophytic branch. The hypothesis of the origin of apicoplasts from the common ancestor of all apicomplexans from plastids of red algae was confirmed.

## 1. Introduction

The rapid growth of the number of sequenced plastid genomes gives rise to assumptions concerning their evolution and regulation not only in algae but also in plastid-bearing non-photosynthetic protists. The latter include the agents of dangerous protozoan infections, malaria and toxoplasmosis. Namely the phylum Apicomplexa includes many parasitic genera. For example, malaria is caused by *Plasmodium* spp.; *Toxoplasma gondii* is one of the most common parasites and can cause toxoplasmosis; *Babesia microti* is the primary cause of human babesiosis. In HIV patients, *Toxoplasma gondii* as well as *Cryptosporidium* spp. can cause serious and often fatal illness. Apicomlexan parasites also cause diseases in animals including cattle, chickens, dogs, and cats.

Apicoplasts are relict nonphotosynthetic plastids found in many species of the supergroup Chromalveolata. They originated from red algae through secondary endosymbiosis. The apicoplast is surrounded by four membranes that could emerge during endosymbiosis. The ancestral genome was reduced by deletions and rearrangements to its present 35 kb size.

Apicoplasts are among the efficient targets for therapeutic intervention and generation of non-virulent strains for rapid vaccine production [1].

All known plastids originate from cyanobacteria [2]. Three branches of primary plastids of independent origin are recognized; they are represented in GenBank by green algae and plants, glaucophyte *Cyanophora paradoxa*, and red algae. At the same time, many species distant from those mentioned above have secondary or tertiary plastids derived from the primary ones. This study is focused on plastids of the rhodophytic branch, which have a common origin with red algal plastids. These comprise apicomplexan apicoplasts [3] as well as plastids of various algae including photosynthetic alveolates [4,5]. The latter include *Durinskia baltica* and *Kryptoperidinium foliaceum* with tertiary plastids originating from the plastids of diatoms, which consequently originate from those of red algae.

All plastid genomes are examples of reductive evolution. The identification of apicoplast origin in non-photosynthetic species is often problematic due to a significant reduction of their genomes. This explains the controversy concerning the origin of apicoplasts [6,7]. Indeed, early reports suggested green algae as the source of apicoplasts. Recent studies confirm that apicoplasts belong to the rhodophytic branch of plastids [3,5]. The identified putative common regulation of gene expression preserved in some apicoplasts is an important argument for the red algal origin of apicoplasts [3]. The coral endosymbiotic algae *Chromera velia* and *Vitrella brassicaformis* share a common ancestry with apicomplexan parasites [8]. A common ancestry of their plastids and apicoplasts can also be anticipated.

Some plastids have no genes of the photosystems and are incapable of photosynthesis but synthesize amino acids and isoprenoids and carry out fatty acid oxidation as well as other chemical reactions. For instance, such plastids are found in red algae *Choreocolax polysiphoniae* (GenBank: NC_026522) [9] or cryptomonad *Cryptomonas paramecium* (GenBank: NC_013703.1), and such apicoplasts are found in many apicomplexan parasites. Comparative analysis of proteomes of photosynthetic and non-photosynthetic species exposes the relationships between different proteins and makes it possible to identify putative regulons of transcription factors encoded in plastids.

Certain apicomplexan species lack apicoplasts, for instance *Cryptosporidium parvum* [10] and *Gregarina niphandrodes* [11,12]. This raises the question of the origin of apicoplasts: do they have a common origin and were lost in some species or were they independently acquired by different groups?

## 2. Materials and Methods

### 2.1. Materials

Plastid genomes were retrieved from GenBank. Table 1 presents the complete list of species and accession numbers of their plastids. These include apicoplasts in Piroplasmida: *Babesia orientalis* strain Wuhan (NC_028029.1) [13], *Babesia microti* strain RI (LK028575.1) [14], *Babesia bovis* T2Bo (NC_011395.1) [15], and *Theileria parva* strain Muguga (NC_007758.1) [16]; in Coccidia: *Eimeria tenella* (NC_004823.1) [17], *Cyclospora cayetanensis* (KP866208.1) [18], and *Toxoplasma gondii* RH (NC_001799.1) [6]; and in Haemosporida: *Leucocytozoon caulleryi* (NC_022667.1) [19] and *Plasmodium chabaudi* [20]. The plastid genome of *Porphyra purpurea* (NC_000925.1) [21] was used as the reference. Note that two variants of plastid genomes in *Plasmodium chabaudi* code for the same proteins but have a different order of genes on the chromosome: “The DNA is present in two different forms A and B that share identical sequence except for the opposite direction of the rRNA/tRNA gene cluster between *rps4* and *sufB*” [20].

**Table 1:** Numbers of proteins (P), clusters (C), and singletons (S) per species.

Locus	Species	P	C	S	Locus	Species	P	C	S
NC_024079.1	*Asterionella formosa*	134	129	0	NC_024084.1	*Leptocylindrus danicus*	132	130	0
NC_024080.1	*Asterionellopsis glacialis*	145	138	1	NC_022667.1	*Leucocytozoon caulleryi*	30	30	0
NC_012898.1	*Aureococcus anophagefferens*	105	105	0	NC_024085.1	*Lithodesmium undulatum*	138	129	0
NC_012903.1	*Aureoumbra lagunensis*	110	110	0	NC_020014.1	*Nannochloropsis gaditana*	119	116	3
NC_011395.1	*Babesia bovis*	32	26	3	NC_022259.1	*N. granulata*	125	123	0
LK028575.1	*B. microti*	31	22	7	NC_022262.1	*N. limnetica*	124	123	0
NC_028029.1	*B. orientalis*	38	28	7	NC_022263.1	*N. oceanica*	126	123	1
NC_021075.1	*Calliarthron tuberculosum*	201	200	1	NC_022260.1	*N. oculata*	126	123	0
NC_025313.1	*Cerataulina daemon*	132	130	0	NC_022261.1	*N. salina*	123	123	0
NC_025310.1	*Chaetoceros simplex*	131	128	0	NC_001713.1	*Odontella sinensis*	140	128	9
NC_020795.1	*Chondrus crispus*	204	204	0	NC_020371.1	*Pavlova lutheri*	111	103	8
NC_026522.1	*Choreocolax polysiphoniae*	71	71	0	NC_016703.2	*Phaeocystis antarctica*	108	108	0
NC_014340.2	*Chromera velia*	78	51	24	NC_021637.1	*P. globosa*	108	108	0
NC_014345.1	*Chromerida* sp. RM11	81	69	5	NC_008588.1	*Phaeodactylum tricornutum*	132	130	0
NC_024081.1	*Coscinodiscus radiatus*	139	130	0	NC_023293.1	*Plasmodium chabaudi*	31	31	0
NC_013703.1	*Cryptomonas paramecium*	82	79	3	NC_017932.1	*P. vivax*	31	31	0
NC_004799.1	*Cyanidioschyzon merolae*	207	189	18	NC_000925.1	*Porphyra purpurea*	209	209	0
NC_001840.1	*Cyanidium caldarium*	197	186	11	NC_023133.1	*Porphyridium purpureum*	224	183	40
KP866208.1	*Cyclospora cayetanensis*	28	27	1	NC_027721.1	*Pseudo-nitzschia multiseries*	104	103	1
NC_024082.1	*Cylindrotheca closterium*	161	142	12	NC_021189.1	*Pyropia haitanensis*	211	210	1
NC_024083.1	*Didymosphenia geminata*	130	128	0	NC_024050.1	*P. perforata*	209	207	2
NC_014287.1	*Durinskia baltica*	129	127	0	NC_007932.1	*P. yezoensis*	209	206	3
NC_013498.1	*Ectocarpus siliculosus*	148	143	1	NC_025311.1	*Rhizosolenia imbricata*	135	123	1
NC_004823.1	*Eimeria tenella*	28	26	2	NC_009573.1	*Rhodomonas salina*	146	145	1
NC_007288.1	*Emiliania huxleyi*	119	112	7	NC_025312.1	*Roundia cardiophora*	140	126	0
NC_024928.1	*Eunotia naegelii*	160	136	2	NC_018523.1	*Saccharina japonica*	139	139	0
NC_015403.1	*Fistulifera solaris*	135	130	1	NC_027589.1	*Teleaulax amphioxeia*	143	143	0
NC_016735.1	*Fucus vesiculosus*	139	139	0	NC_014808.1	*Thalassiosira oceanica*	142	126	1
NC_024665.1	*Galdieria sulphuraria*	182	181	1	NC_008589.1	*T. pseudonana*	141	127	0
NC_023785.1	*Gracilaria salicornia*	202	200	2	NC_025314.1	*T. weissflogii*	141	127	0
NC_006137.1	*G. tenuistipitata*	203	201	2	NC_007758.1	*Theileria parva*	44	27	12
NC_021618.1	*Grateloupia taiwanensis*	233	201	32	NC_001799.1	*Toxoplasma gondii*	26	21	5
NC_000926.1	*Guillardia theta*	147	142	5	NC_026851.1	*Trachydiscus minutus*	137	124	8
NC_010772.1	*Heterosigma akashiwo*	156	139	3	NC_027746.1	*Triparma laevis*	141	135	4
NC_014267.1	*Kryptoperidinium foliaceum*	139	132	6	NC_016731.1	*Ulnaria acus*	130	128	0
NC_027093.1	*Lepidodinium chlorophorum*	62	52	7	NC_026523.1	*Vertebrata lanosa*	192	191	1

### 2.2. Methods

Bacterial-type promoters were identified using the method described elsewhere [22,23] based on the data relating nucleotide substitutions with the intensity of binding of bacterial-type RNA polymerase to the promoter upstream of the *psbA* gene in mustard plastids [24]. On the whole this method relies on comparison of genome regions with known promoters. The *sfdp* program of the *Graphviz* package [25] was used to visualize the clusters (protein families). The sequence Logos were prepared with WebLogo tool [26]. The phylogenetic trees were visualized using the MEGA 6 [27] and TreeView 1.6.6 [28] software. Conserved protein domains were identified using the Pfam database [29]. Amino acid sequences were aligned using the MUSCLE algorithm [30]. Trees were generated from multiple alignments of protein sequences using the RAxML software [31].

Protein clustering was done with the method from [32] and successfully tested in a series of works [33,34,35]. Let us note that MCL [36] is commonly used to define clusters in a graph. However, our method performs well as confirmed by correct clusterings obtained by this method for reference data [33,34,35]; at the same time, it requires essentially less computation time.

The representation of proteins as points in Euclidean space makes it possible to apply clustering methods described in [37,38,39,40,41]. However, the real data on proteins are inconsistent with the Euclidean metric. Our approach to clustering does not require even the triangle inequality to hold.

In mathematical terms, the following problem is solved. We are given a set of protein sequences. It is required to generate a clustering, *i.e.*, to partition this set into pairwise disjoint subsets so that a cluster includes proteins with similar sequences from different proteomes, and proteins from the same proteome are included in the same cluster as rarely as possible.

### 2.3. Description of the Clustering Algorithm

We are given a set of proteomes *S_i_* and sets of component proteins *P_ij_* for each proteome. The BLAST raw score was used to compute the similarity *s*_o_(*P*_1_,*P*_2_) between proteins; *s*_o_(*P_ij_*,*P_kl_*) is evaluated for all pairs of proteins (*P_ij_*,*P_kl_*) from all pairs of proteomes, so that the normalized similarity can be computed:

$$s(P_{ij},P_{kl})=2s_{0}(P_{ij},P_{kl}){\left(s_{0}(P_{ij},P_{ij})+s_{0}(P_{kl},P_{kl})\right)}^{-1}$$

It peaks for identical proteins. Let us consider an undirected graph *G*_o_ with a set of nodes {*P_ij_*}, which are connected by an edge if the BLAST *E*-value for the corresponding pair of proteins is no less than the expect threshold. Each edge (*P_ij_*,*P_kl_*) is given the value *s*(*P_ij_*,*P_kl_*), which will be referred to as the edge *weight*; loops are not allowed. *G*_o_ is used to generate a sparse graph *G* which only includes edges meeting the following requirements:

$$s(P_{ij},P_{kl})=\underset{m}{\max}\;s(P_{im},P_{kl})=\underset{m}{\max}\;s(P_{ij},P_{km})\;\mathrm{and}\;s(P_{ij},P_{kl})\geq L$$

where the maximums are taken for all proteins of the corresponding plastids *i* and *k*, and *L* is the algorithm parameter. The case when *i* = *k* imposes the constraint that *m* ≠ *l* and the second equality is not considered.

Our algorithm implements Kruskal’*s* procedure [42] for the graph *G* to generate a forest *F* (an acyclic subgraph with trees as the connected components) that includes all nodes from *G*. Specifically, edges in *G* are searched in descending order of their weight (in the case of equal weights, the edges connecting proteins of the same proteome are considered first), and the edges from *G* whose addition to *F* do not introduce a cycle in *F* are called edges of the constructed forest *F*. Total weight of all edges in the forest is called its *weight*. The weight of the resulting forest is the highest among all other forests in *G*.

The following procedure of forest partition generating a set *C* of desired protein clusters is applied to the forest *F*. Let *T* be a tree from *F* and *e* be the edge in *T* with the minimum weight *s* among all edges in *T*. If *s* < *H*, where *H* is the algorithm parameter, and *T* does not meet the *criterion of tree preservation* stated below, then *T* is replaced in *F* with two new trees *F*’ and *F*” by removing the edge *e* from *T*; otherwise (when the criterion is met or *s* ≥ *H*) the tree *T* is transposed to the set *C*.

The criterion of tree *T* preservation is that two conditions are satisfied: (1) the edge (*P_ij_*, *P_kl_*) with the minimum weight in *T* connects proteins *P_ij_* and *P_kl_*, where *i* ≠ *k*; and (2) any pair of nodes *P_ij_* and *P_il_* in the tree *T* corresponding to proteins of plastid *i* is connected in *T* by a path composed of nodes that correspond to proteins of this plastid.

If there are trees remaining in *F*, the next tree *T* in *F* is considered; otherwise the algorithm terminates. The resulting set of trees *C* represents clusters of initial proteins: each cluster consists of sequences assigned to all nodes of the same tree.

The following algorithm parameters were used: *H* = 0.60, *E* = 0.001, and *L* = 0.

## 3. Results

### 3.1. Clustering of Proteins

We have clustered proteins encoded in the plastids of the rhodophytic branch. The results are publicly available at http://lab6.iitp.ru/ppc/redline72/. The database functions allow rapid cluster identification by either a fragment of a protein amino acid sequence or by a protein phylogenetic profile.

The total number of proteins is 9286; the number of singletons is 265; and the number of clusters is 305. The number of clusters including exactly *n* proteins in a particular species and no more than *n* proteins in any species is referred to as PC(*n*). For this clustering, PC(1) = 223, PC(2) = 79, PC(3) = 2, and PC(4) = 1. Some general data about the clusters are given in Table 1.

**Figure 1:**
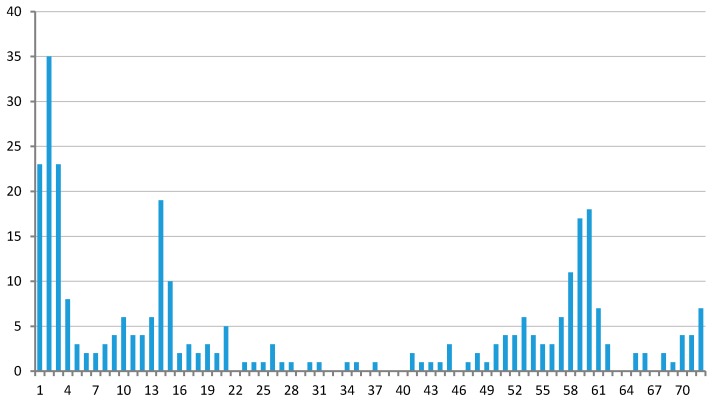
Dependence of the number of clusters on the number of species represented in it.

**Figure 2:**
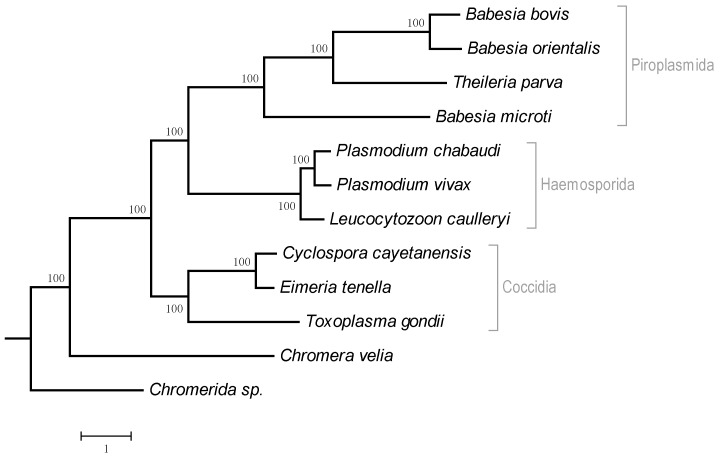
Tree of apicoplasts. *Chromera velia* and *Chromerida* sp. RM11 plastids were used as the outgroup.

The relationship between the number of clusters and the number of species in them is shown in Figure 1. Only seven clusters are represented in all considered plastids: six ribosomal proteins L2, L14, L16, S3, S11, and S12 as well as RNA polymerase beta subunit (RpoB). The genes *odpA* (*pdhA*), *odpB* (*pdhB*), *trpA*, *trpG*, *tilS* (*ycf62*), and *infC* are specific for all plastids in the considered Rhodophyta species. The tree of apicoplasts and plastids of photosynthetic *Chromera velia* and *Chromerida* sp. generated from the concatenated multiple alignment of proteins is shown in Figure 2.

**Figure 3:**
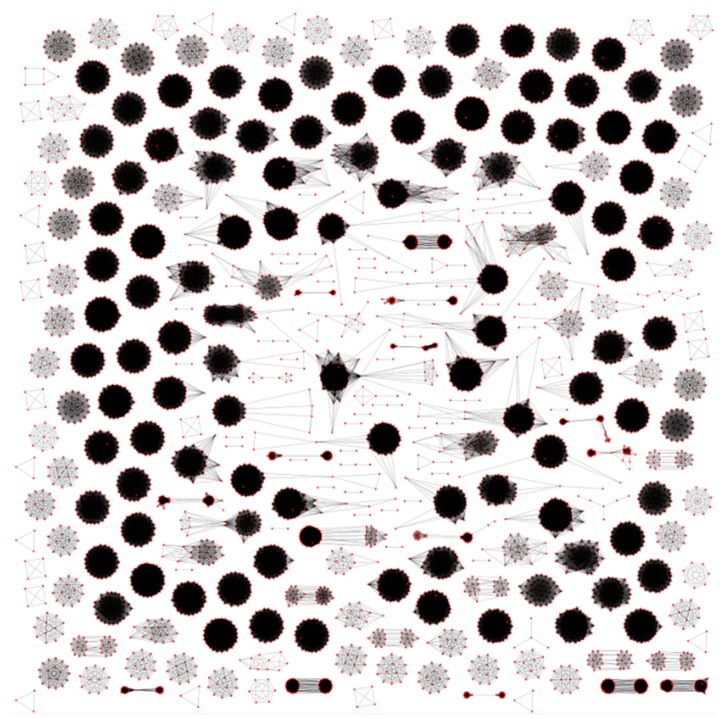
Connectivity components of the sparse graph of proteins. Red dots represent proteins and lines represent bidirectional BLAST hits.

Figure 3 exemplifies a sparse graph *G* for our data. Many connected components are high density or even cliques. The graph contains 9072 non-isolated nodes and 223,377 edges; 245 isolated nodes (singletons) in it are due to the absence of bidirectional best hits for the corresponding proteins, and only 20 singletons were added by the algorithm during tree partitioning. The number of connected components of the sparse graph excluding singletons is 33 less than the ultimate number of clusters generated by the algorithm. Finding them is a non-trivial result of our algorithm.

Let us specify the following connected components of the sparse graph that are partitioned into smaller clusters by our algorithm: ApcA+ApcD, ApcB+ApcF, ApcE+CpcG, AtpA+AtpB, CarA+TrpG, CbbX(CfxQ)+FtsH+Ycf46, ChlB+ChlN, CpcB+CpeB, InfB+TufA, OdpA+IlvB, PetJ+PsbV, PsaA+PsaB, PsbA+PsbD, PsbB+PsbC, PsbK+Rpl20, RpoC1+RpoC2+RpoC2B, Rps4+Ycf24(SufB), Rpl22+Ycf88, Ycf3+Ycf37, Ycf27(OmpR)+Ycf29, and Ycf60+Ycf90. Each connected component here is denoted by a typical protein name; non-orthologous proteins are separated by the plus sign.

### 3.2. Regulons of Transcription Factors Encoded by Plastids

As compared to our previous data [35], the clusters of the MoeB and Ycf28 proteins were both supplemented by proteins encoded in the plastids of *Vertebrata lanosa*; neither of these proteins is encoded in plastids of *Choreocolax polysiphoniae* or any species beyond Rhodophyta. The profile identical to that of MoeB and Ycf28 was found in the proteins encoded by the *apcA*, *apcB*, *apcD*, *apcE*, *apcF*, *carA*, *cpcA*, *cpcB*, *cpcG*, *gltB*, *nblA* (*ycf18*), *preA*, and *rpl28* genes; however, their 5’-leader sequences lack the conserved site found upstream of the *moeB* genes instead of the typical -35 promoter box.

**Figure 4:**
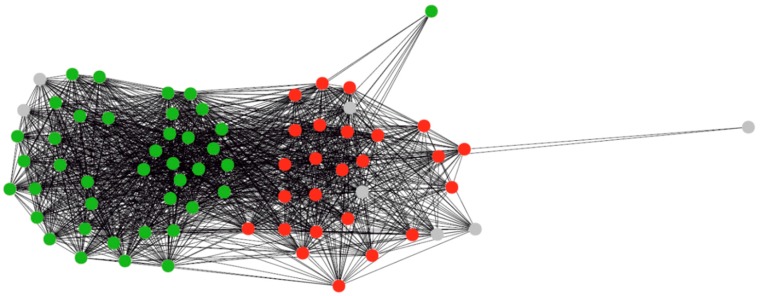
Graph of Ycf19 and Ycf89 proteins. Colors indicate the proteins annotation: red = Ycf19, green = Ycf89, gray = no name specified.

The transcription factor Ycf29 is encoded in plastids of cryptomonads and rhodophytic algae except *Porphyridium purpureum*. The Ycf29 proteins are listed in Table 2. In the sparse graph, the Ycf29 and Ycf27 (OmpR) proteins belonged to the same connected component but were separated after clustering by our algorithm, which corresponds to the NCBI annotation. No other proteins with such phylogenetic profile have been identified. A similar profile was observed for the CemA protein found in *Porphyridium purpureum* but not in *Choreocolax polysiphoniae*. CemA includes the PF03040 domain and was localized to the inner face of the outer membrane in chloroplasts but not to the thylakoid membrane. Cyanobacterial proteins orthologous to CemA are involved in carbon dioxide transport but are not transporters [43]. The membrane protein Ycf19 also has a similar phylogenetic profile. A sequence close to the consensus of the conserved bacterial-type promoter was found upstream of the *ycf19* gene. Since Ycf29 is a part of the two-component signaling system, its regulon is linked to the response to environmental rather than intraplastid changes. The Ycf19 and Ycf89 proteins are not partitioned with the clustering parameters used. At the same time, the proteins listed in Ycf19 annotations together with several related proteins constitute a dense subgraph. The graph of proteins Ycf19 and Ycf89 generated by the algorithm is shown in Figure 4.

**Table 2:** Ycf29 proteins encoded in the plastids of the rhodophytic branch.

Accession	Source	Protein Description
YP_007878178.1	*Calliarthron tuberculosum*	conserved hypothetical plastid protein
YP_007627336.1	*Chondrus crispus*	conserved hypothetical plastid protein
YP_009122074.1	*Choreocolax polysiphoniae*	hypothetical protein
YP_003359295.1	*Cryptomonas paramecium*	TctD-like protein
NP_849011.1	*Cyanidioschyzon merolae*	ompR-like transcriptional regulator
NP_045122.1	*Cyanidium caldarium*	regulatory component of sensory transduction system
YP_009051025.1	*Galdieria sulphuraria*	putative transcriptional regulator LuxR
YP_009019567.1	*Gracilaria salicornia*	tctD transcriptional regulator
YP_063559.1	*Gracilaria tenuistipitata*	tctD transcriptional regulator
YP_008144796.1	*Grateloupia taiwanensis*	putative transcriptional regulator Ycf29
NP_050668.1	*Guillardia theta*	tctD homolog
NP_053953.1	*Porphyra purpurea*	ORF29
YP_007947873.1	*Pyropia haitanensis*	hypothetical chloroplast protein 29
YP_009027627.1	*Pyropia perforata*	hypothetical chloroplast protein 29
YP_537024.1	*Pyropia yezoensis*	hypothetical chloroplast protein 29
YP_001293481.1	*Rhodomonas salina*	TctD-like protein
YP_009159161.1	*Teleaulax amphioxeia*	TctD-like protein
YP_009122313.1	*Vertebrata lanosa*	hypothetical protein

### 3.3. Regulation of Ycf24 (SufB) Translation Initiation

**Figure 5:**
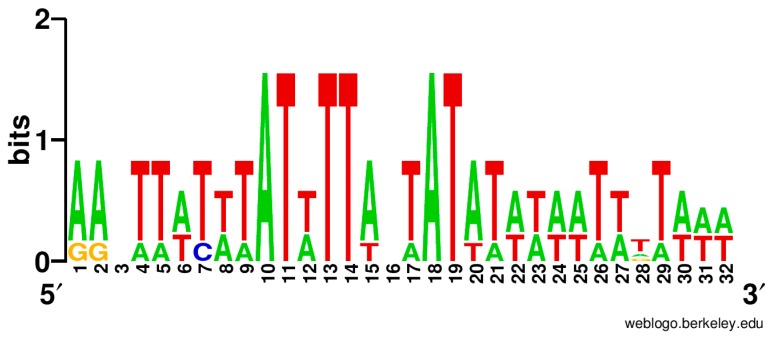
Sequence LOGO of the putative site in the 5’-untranslated region of *ycf24* (*sufB*).

A conserved site was found in the 5’-untranslated region of *ycf24* (*sufB*) in *Eimeria tenella*, *Cyclospora cayetanensis*, *Toxoplasma gondii* RH, *Leucocytozoon caulleryi*, *Plasmodium* chabaudi, and *Porphyra purpurea*. The sequence logo of this site is shown in Figure 5.

### 3.4. Regulation of Translation Initiation in Babesia spp. and Theileria parva 

**Figure 6:**
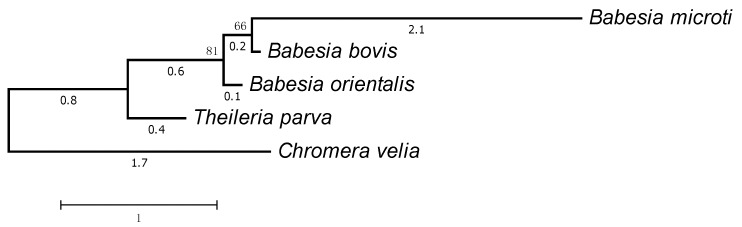
The tree of proteins encoded by the plastid genes located between the *rpl14* and *rps8* genes in *Babesia* spp. and *Theileria parva*. The plastid protein L5 from *Chromera velia* was used as the outgroup.

The genes from plastids of the Piroplasmida order lying between the *rpl14* and *rps8* are of particular interest. Although one such gene codes for the ribosomal protein L5 in many plastid-bearing algae of the rhodophytic branch, *rpl14* and *rps8* are neighboring genes in Coccidia and Haemosporida. The functional identification of the protein encoded by the gene lying between *rpl14* and *rps8* is questionable. Our clustering in *Babesia bovis*, *Babesia orientalis*, and *Theileria parva* suggests that this gene codes for the ribosomal protein L5, which belongs to a large cluster. In *Babesia microti*, this protein forms a singleton but is also annotated as L5. At the same time, it is only marginally similar to ribosomal proteins according to Pfam. The tree of these proteins is shown in Figure 6, and the proteins are listed in Table 3.

Conserved sites were identified in the leader regions 170–100 nt upstream of such genes in Piroplasmida. In *Babesia* spp., such sites reside within the coding sequence of *rpl14*. However, there was an insertion near the site, which is missing in orthologous L14 proteins. The sequence of this insertion is TSYSIDDRNRFKD in *Babesia bovis*. In *Theileria parva*, the site is not overlapped by the coding sequences. The corresponding transcription factor remains unknown in this case.

**Table 3:** The plastid proteins in Piroplasmida with a marginal similarity with the ribosomal protein L5 discussed in Section 3.4 and Section 4.4 are shown.

Accession	Source	Protein Description
YP_002290869.1	*Babesia bovis*	hypothetical protein
CDR32594.1	*Babesia microti*	ribosomal protein L5
YP_009170371.1	*Babesia orientalis*	ribosomal protein L5
XP_762679.1	*Theileria parva*	hypothetical protein

## 4. Discussion

### 4.1. Protein Clustering

Overall, the data obtained indicate a good agreement between the clustering of plastid-encoded proteins performed by our algorithm and published data on the protein and species evolution. The proposed clustering algorithm and its software implementation are applicable to a wide range of problems related to graphs.

The clustering pattern of proteins encoded in red algal plastids demonstrate a substantial distance of *Porphyridium purpureum* from other species, which is accompanied by multiple DNA rearrangements in Rhodophyta plastids [44]; in addition, it demonstrates the separation of the Cyanidiaceae family including *Galdieria sulphuraria*, *Cyanidium caldarium*, and *Cyanidioschyzon merolae*.

### 4.2. Regulons of Plastid-Encoded Transcription Factors Ycf28, Ycf29, and Ycf30

The coincidence of the phylogenetic profiles of Ycf28 and MoeB reported previously [35] has been confirmed. The Ycf28 protein demonstrates a significant similarity with the cyanobacterial transcription factor NtcA. Consequently, we propose that Ycf28 is the factor that controls the transcription of the *moeB* gene by binding the DNA region near the promoter where the conserved motif was identified. There are no grounds to believe that Ycf28 is related to nitrogen metabolism, which assumes a change of the transcription factor specificity relative to cyanobacteria contrary to the previous proposal [45]. The absence of the typical -35 promoter box upstream of the *moeB* gene indicates that Ycf28 is a transcription activator.

The presence of Ycf29 in the plastid genomes of non-photosynthetic *Cryptomonas paramecium* and *Choreocolax polysiphoniae* indicates that this protein regulates processes related to photosynthesis. One can assume that Ycf19 orthologs include proteins in the large cluster combining Ycf19 and Ycf89 that are encoded in plastids together with the Ycf29 factor. This allows us to refine protein clustering and, at the same time, to identify the putative photosynthesis-independent regulation.

Plastids of many algal species are known to encode the transcription factor Ycf30, which controls the expression of the *rbcLS* genes coding for subunits of ribulose-bisphosphate carboxylase (EC 4.1.1.39) as well as of the *cbbX* gene. Light-induced transcriptional activation was experimentally demonstrated and the Ycf30-binding motif was identified in these genes in plastids isolated from *Cyanidioschyzon merolae* [46]. Our phylogenetic profiles of these proteins agree with these data. However, the variability of Ycf30-binding site complicates its unambiguous identification in the DNA sequence. The sequence variability of experimentally confirmed Ycf30-binding site suggests that the factor binding to DNA largely depends on the DNA curvature [47] or electrostatic potential along the DNA [48] rather than on the nucleotide context.

### 4.3. Regulation of Ycf24 (SufB) Translation Initiation

The same regulation found in red algae, Coccidia, and Haemosporida supports the common origin of all apicoplasts from red algal plastids. Moreover, early separation of these apicomplexan groups naturally suggests that *Cryptosporidium* spp. and *Gregarina niphandrodes* lost their apicoplasts in the course of evolution but the common ancestor of apicomplexans had apicoplasts.

Moreover, the site identical to that upstream of *ycf24* was found in the 5’-untranslated region of *rps4* of *Toxoplasma gondii* [3]. This indicates possible the common regulation of translation in the apicoplast.

### 4.4. Regulation of Translation Initiation in Babesia spp. and Theileria parva

We believe that the gene coding for the ribosomal protein L5 was eliminated from the apicoplast in the ancestor of apicomplexan parasites, and a new gene was inserted into this chromosomal locus in the ancestor of Piroplasmida. The recognition of a new type of proteins is confirmed by the analysis of their 5’-leader regions, where conserved sites were identified. Indeed, it is natural to assume that a conserved site is involved in the regulation of gene expression, and the same expression pattern indicates a common functional significance of the corresponding proteins.

## 5. Conclusions

We have made a publicly available web service for protein identification by their phylogenetic profile. To our knowledge, no other services for the identification of plastid-encoded proteins by their phylogenetic profile (the two lists of species) are available. Our method allowed us to confirm the previous assumption concerning the regulation of plastid gene expression in the rhodophytic branch. In particular, our results confirm the hypothesis that apicoplasts in the common ancestor of apicomplexans descend from red algal plastids.
